# *N*-[(*E*)-4-Chloro­benzyl­idene]-2,3-dimethyl­aniline

**DOI:** 10.1107/S1600536810020933

**Published:** 2010-06-05

**Authors:** M. Nawaz Tahir, Muhammad Ilyas Tariq, Shahbaz Ahmad, Muhammad Sarfraz, Abdul Qayyum Ather

**Affiliations:** a Department of Physics, University of Sargodha, Sargodha, Pakistan; b Department of Chemistry, University of Sargodha, Sargodha, Pakistan; c Applied Chemistry Research Center, PCSIR Laboratories complex, Lahore 54600, Pakistan

## Abstract

In the title compound, C_15_H_14_ClN, the conformation about the C=N bond is *trans* and the dihedral angle between the aromatic rings is 51.48 (4)°. In the crystal, some very weak C—H⋯π inter­actions may help to establish the packing.

## Related literature

For a related structure and background to Schiff bases, see: Tariq *et al.* (2010 ▶). For related structures with different substituents at the N-bonded ring, see: Bürgi *et al.* (1968 ▶); Kazak *et al.* (2004 ▶); Ojala *et al.* (2001 ▶).
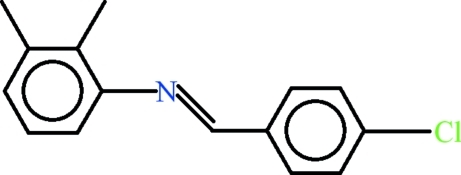


## Experimental

### 

#### Crystal data

C_15_H_14_ClN
*M* *_r_* = 243.72
Monoclinic, 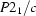

*a* = 12.8981 (4) Å
*b* = 7.7999 (2) Å
*c* = 15.0449 (5) Å
β = 119.315 (2)°
*V* = 1319.75 (7) Å^3^
*Z* = 4
Mo *K*α radiation
μ = 0.27 mm^−1^
*T* = 296 K
0.30 × 0.20 × 0.20 mm

#### Data collection

Bruker Kappa APEXII CCD diffractometer
Absorption correction: multi-scan (*SADABS*; Bruker, 2005 ▶) *T*_min_ = 0.939, *T*_max_ = 0.950
10119 measured reflections
2378 independent reflections
1722 reflections with *I* > 2σ(*I*)
*R*_int_ = 0.026

#### Refinement

*R*[*F* ^2^ > 2σ(*F* ^2^)] = 0.041
*wR*(*F* ^2^) = 0.116
*S* = 1.05
2378 reflections
157 parameters
H-atom parameters constrained
Δρ_max_ = 0.15 e Å^−3^
Δρ_min_ = −0.19 e Å^−3^

### 

Data collection: *APEX2* (Bruker, 2007 ▶); cell refinement: *SAINT* (Bruker, 2007 ▶); data reduction: *SAINT*; program(s) used to solve structure: *SHELXS97* (Sheldrick, 2008 ▶); program(s) used to refine structure: *SHELXL97* (Sheldrick, 2008 ▶); molecular graphics: *ORTEP-3* (Farrugia, 1997 ▶) and *PLATON* (Spek, 2009 ▶); software used to prepare material for publication: *WinGX* (Farrugia, 1999 ▶) and *PLATON* (Spek, 2009 ▶).

## Supplementary Material

Crystal structure: contains datablocks global, I. DOI: 10.1107/S1600536810020933/hb5479sup1.cif

Structure factors: contains datablocks I. DOI: 10.1107/S1600536810020933/hb5479Isup2.hkl

Additional supplementary materials:  crystallographic information; 3D view; checkCIF report

## Figures and Tables

**Table 1:** Hydrogen-bond geometry (Å, °) *Cg*1 and *Cg*2 are the centroids of the C1–C6 and C10–C15 benzene rings, respectively.

*D*—H⋯*A*	*D*—H	H⋯*A*	*D*⋯*A*	*D*—H⋯*A*
C6—H6⋯*Cg*1^i^	0.93	2.99	3.649 (2)	129
C7—H7*A*⋯*Cg*2^ii^	0.96	2.93	3.757 (3)	145
C12—H12⋯*Cg*1^iii^	0.93	2.96	3.793 (3)	150
C7—H7*E*⋯*Cg*2^ii^	0.96	3.00	3.757 (3)	137

Symmetry codes: (i) 
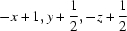
; (ii) 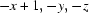; (iii) 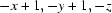.

**Fig. 1.**
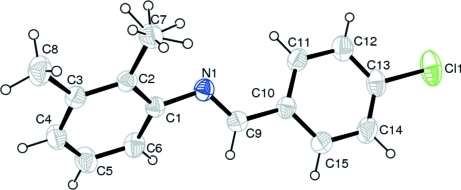
View of (I) with displacement ellipsoids drawn at the 30% probability level.

## supplementary crystallographic information

### Comment

In continuation to synthesize various Schiff bases (Tariq *et al.*, 2010) of 2,3-dimethylaniline, the title compound (I, Fig. 1) is being reported.

The crystal structure of *p*-chlorobenzylideneaniline (Bürgi, *et al.*, 1968), *p*-cyano-*N*-(*p*-chlorobenzylidene)aniline (Ojala *et al.*, 2001) and 4-((4-Chlorobenzylidene)amino)phenol (Kazak *et al.*, 2004) have been published which contain the chloro group at *para* position. The title compound differs from these due to substitutions at the aniline.

In (I), the 2,3-dimethylanilinic group A (C1—C8/N1) and the *p*-chlorobenzaldehyde B (C9—C15/CL1) are planar with maximum r. m. s. deviations of 0.0121 and 0.0071 Å, respectively. The dihedral angle between A/B is 51.48 (4)°. The molecules are essentially monomer with no appreciable intra-molecular H-bonding. The phenyl ring of 2,3-dimethylaniline has longer bond length [1.375 (3)–1.399 (2) Å] as compared to the phenyl ring of *p*-chlorobenzaldehyde [1.364 (4)–1.386 (3) Å]. The observed value of C═N bond is 1.264 (3) Å. All these bond lengths are compareable with 2,3-dimethyl-*N*-[(*E*)-(4-nitrophenyl)methylidene]aniline (Tariq *et al.*, 2010). The molecules are stabilized due to C—H···π interactions (Table 1). The H-atoms of the methyl at *ortho* position are disordered over two set of sites with occupancy ratio 0.60 (3):0.40 (3).

### Experimental

Equimolar quantities of 2,3-dimethylaniline and 4-chlorobenzaldehyde were refluxed in methanol for 45 min resulting in yellow solution. The solution was kept at room temperature which affoarded colourless prisms of (I) after 48 h.

### Refinement

All H-atoms were positioned geometrically (C–H = 0.93, 0.96 Å) and refined as riding with *U*_iso_(H) = *xU*_eq_(C), where *x* = 1.2 for aryl and *x* = 1.5 for methyl H-atoms. From the observation of difference Fourier map, it was concluded that H-atoms of one of the *ortho* methyl are disordered.

### Crystal data

**Table:**

C_15_H_14_ClN	*F*(000) = 512
*M**_r_* = 243.72	*D*_x_ = 1.227 Mg m^−^^3^
Monoclinic, *P*2_1_/*c*	Mo *K*α radiation, λ = 0.71073 Å
Hall symbol: -P 2ybc	Cell parameters from 1467 reflections
*a* = 12.8981 (4) Å	θ = 2.3–25.3°
*b* = 7.7999 (2) Å	µ = 0.27 mm^−^^1^
*c* = 15.0449 (5) Å	*T* = 296 K
β = 119.315 (2)°	Prism, colourless
*V* = 1319.75 (7) Å^3^	0.30 × 0.20 × 0.20 mm
*Z* = 4	

### Data collection

**Table:**

Bruker Kappa APEXII CCD diffractometer	2378 independent reflections
Radiation source: fine-focus sealed tube	1722 reflections with *I* > 2σ(*I*)
graphite	*R*_int_ = 0.026
Detector resolution: 8.10 pixels mm^-1^	θ_max_ = 25.3°, θ_min_ = 2.7°
ω scans	*h* = −15→15
Absorption correction: multi-scan (*SADABS*; Bruker, 2005)	*k* = −8→9
*T*_min_ = 0.939, *T*_max_ = 0.950	*l* = −18→18
10119 measured reflections	

### Refinement

**Table:**

Refinement on *F*^2^	Primary atom site location: structure-invariant direct methods
Least-squares matrix: full	Secondary atom site location: difference Fourier map
*R*[*F*^2^ > 2σ(*F*^2^)] = 0.041	Hydrogen site location: inferred from neighbouring sites
*wR*(*F*^2^) = 0.116	H-atom parameters constrained
*S* = 1.05	*w* = 1/[σ^2^(*F*_o_^2^) + (0.0502*P*)^2^ + 0.2848*P*] where *P* = (*F*_o_^2^ + 2*F*_c_^2^)/3
2378 reflections	(Δ/σ)_max_ < 0.001
157 parameters	Δρ_max_ = 0.15 e Å^−^^3^
0 restraints	Δρ_min_ = −0.19 e Å^−^^3^

### Special details

**Table:**

Geometry. Bond distances, angles *etc*. have been calculated using the rounded fractional coordinates. All su's are estimated from the variances of the (full) variance-covariance matrix. The cell e.s.d.'s are taken into account in the estimation of distances, angles and torsion angles
Refinement. Refinement of *F*^2^ against ALL reflections. The weighted *R*-factor *wR* and goodness of fit *S* are based on *F*^2^, conventional *R*-factors *R* are based on *F*, with *F* set to zero for negative *F*^2^. The threshold expression of *F*^2^ > σ(*F*^2^) is used only for calculating *R*-factors(gt) *etc*. and is not relevant to the choice of reflections for refinement. *R*-factors based on *F*^2^ are statistically about twice as large as those based on *F*, and *R*- factors based on ALL data will be even larger.

### Fractional atomic coordinates and isotropic or equivalent isotropic displacement parameters (Å^2^)

**Table:**

	*x*	*y*	*z*	*U*_iso_*/*U*_eq_	Occ. (<1)
Cl1	0.03504 (6)	0.38426 (9)	−0.38533 (4)	0.1078 (3)	
N1	0.47950 (12)	0.29383 (18)	0.09822 (10)	0.0549 (5)	
C1	0.55065 (14)	0.2685 (2)	0.20499 (12)	0.0492 (5)	
C2	0.66149 (14)	0.1905 (2)	0.24128 (12)	0.0502 (5)	
C3	0.73394 (15)	0.1691 (2)	0.34649 (13)	0.0550 (6)	
C4	0.69544 (17)	0.2286 (3)	0.41183 (14)	0.0673 (7)	
C5	0.58704 (18)	0.3083 (3)	0.37546 (14)	0.0762 (8)	
C6	0.51425 (17)	0.3277 (3)	0.27191 (14)	0.0654 (6)	
C7	0.70003 (18)	0.1280 (3)	0.16739 (15)	0.0750 (8)	
C8	0.85264 (17)	0.0805 (3)	0.39021 (15)	0.0821 (8)	
C9	0.36888 (15)	0.2672 (2)	0.05682 (13)	0.0568 (6)	
C10	0.28740 (15)	0.2988 (2)	−0.05166 (13)	0.0555 (6)	
C11	0.32577 (17)	0.3762 (2)	−0.11338 (14)	0.0642 (7)	
C12	0.24792 (19)	0.4016 (3)	−0.21581 (15)	0.0729 (7)	
C13	0.13238 (18)	0.3509 (3)	−0.25623 (14)	0.0691 (7)	
C14	0.09207 (17)	0.2742 (3)	−0.19726 (16)	0.0848 (9)	
C15	0.16989 (16)	0.2490 (3)	−0.09440 (15)	0.0753 (8)	
H4	0.74368	0.21437	0.48175	0.0807*	
H5	0.56297	0.34907	0.42059	0.0914*	
H6	0.44060	0.38061	0.24705	0.0784*	
H7A	0.68581	0.00694	0.15699	0.1125*	0.60 (3)
H7B	0.78330	0.15045	0.19446	0.1125*	0.60 (3)
H7C	0.65559	0.18672	0.10356	0.1125*	0.60 (3)
H8A	0.88586	0.06855	0.46260	0.1231*	
H8B	0.90540	0.14723	0.37604	0.1231*	
H8C	0.84233	−0.03092	0.35984	0.1231*	
H9	0.33782	0.22566	0.09688	0.0681*	
H11	0.40448	0.41135	−0.08561	0.0771*	
H12	0.27416	0.45295	−0.25702	0.0875*	
H14	0.01323	0.23925	−0.22586	0.1018*	
H15	0.14274	0.19794	−0.05375	0.0904*	
H7D	0.63260	0.08266	0.10781	0.1125*	0.40 (3)
H7E	0.75885	0.03979	0.19898	0.1125*	0.40 (3)
H7F	0.73326	0.22166	0.14822	0.1125*	0.40 (3)

### Atomic displacement parameters (Å^2^)

**Table:**

	*U*^11^	*U*^22^	*U*^33^	*U*^12^	*U*^13^	*U*^23^
Cl1	0.1029 (5)	0.1121 (5)	0.0596 (4)	0.0316 (4)	0.0019 (3)	−0.0013 (3)
N1	0.0515 (8)	0.0532 (9)	0.0526 (8)	0.0013 (7)	0.0197 (7)	0.0026 (7)
C1	0.0506 (9)	0.0446 (9)	0.0496 (9)	−0.0022 (7)	0.0223 (8)	−0.0008 (7)
C2	0.0496 (9)	0.0472 (9)	0.0521 (9)	−0.0034 (7)	0.0237 (8)	−0.0011 (8)
C3	0.0507 (10)	0.0530 (10)	0.0547 (10)	−0.0025 (8)	0.0208 (8)	0.0002 (8)
C4	0.0662 (12)	0.0780 (14)	0.0478 (10)	−0.0004 (10)	0.0203 (9)	−0.0031 (10)
C5	0.0761 (14)	0.0942 (16)	0.0587 (12)	0.0091 (12)	0.0333 (10)	−0.0150 (11)
C6	0.0565 (10)	0.0693 (12)	0.0634 (11)	0.0110 (9)	0.0240 (9)	−0.0071 (10)
C7	0.0641 (12)	0.1002 (16)	0.0640 (12)	0.0125 (11)	0.0339 (10)	−0.0014 (11)
C8	0.0608 (12)	0.0989 (17)	0.0680 (12)	0.0163 (11)	0.0172 (10)	0.0052 (12)
C9	0.0553 (11)	0.0592 (11)	0.0562 (10)	−0.0001 (8)	0.0276 (9)	0.0001 (9)
C10	0.0517 (10)	0.0541 (10)	0.0549 (10)	0.0030 (8)	0.0215 (8)	−0.0039 (8)
C11	0.0590 (11)	0.0613 (12)	0.0598 (11)	−0.0094 (9)	0.0193 (9)	0.0031 (9)
C12	0.0848 (14)	0.0598 (12)	0.0607 (11)	−0.0062 (10)	0.0253 (11)	0.0057 (9)
C13	0.0657 (12)	0.0668 (13)	0.0553 (11)	0.0151 (10)	0.0145 (10)	−0.0052 (10)
C14	0.0459 (10)	0.122 (2)	0.0722 (14)	0.0036 (12)	0.0178 (10)	−0.0179 (14)
C15	0.0529 (11)	0.1084 (18)	0.0644 (12)	−0.0042 (11)	0.0285 (10)	−0.0059 (12)

### Geometric parameters (Å, °)

**Table:**

Cl1—C13	1.740 (2)	C4—H4	0.9300
N1—C1	1.421 (2)	C5—H5	0.9300
N1—C9	1.264 (3)	C6—H6	0.9300
C1—C2	1.396 (3)	C7—H7A	0.9600
C1—C6	1.382 (3)	C7—H7B	0.9600
C2—C3	1.399 (2)	C7—H7C	0.9600
C2—C7	1.504 (3)	C7—H7D	0.9600
C3—C4	1.381 (3)	C7—H7E	0.9600
C3—C8	1.506 (3)	C7—H7F	0.9600
C4—C5	1.375 (3)	C8—H8A	0.9600
C5—C6	1.378 (3)	C8—H8B	0.9600
C9—C10	1.466 (2)	C8—H8C	0.9600
C10—C11	1.386 (3)	C9—H9	0.9300
C10—C15	1.381 (3)	C11—H11	0.9300
C11—C12	1.381 (3)	C12—H12	0.9300
C12—C13	1.364 (4)	C14—H14	0.9300
C13—C14	1.366 (3)	C15—H15	0.9300
C14—C15	1.386 (3)		
			
C1—N1—C9	118.88 (16)	C5—C6—H6	120.00
N1—C1—C2	118.29 (16)	C2—C7—H7A	109.00
N1—C1—C6	121.03 (17)	C2—C7—H7B	109.00
C2—C1—C6	120.59 (16)	C2—C7—H7C	109.00
C1—C2—C3	118.90 (17)	C2—C7—H7D	109.00
C1—C2—C7	119.88 (15)	C2—C7—H7E	109.00
C3—C2—C7	121.21 (18)	C2—C7—H7F	109.00
C2—C3—C4	119.44 (18)	H7A—C7—H7B	109.00
C2—C3—C8	121.46 (17)	H7A—C7—H7C	109.00
C4—C3—C8	119.10 (16)	H7B—C7—H7C	109.00
C3—C4—C5	121.24 (17)	H7D—C7—H7E	109.00
C4—C5—C6	119.8 (2)	H7D—C7—H7F	109.00
C1—C6—C5	120.0 (2)	H7E—C7—H7F	109.00
N1—C9—C10	122.83 (18)	C3—C8—H8A	109.00
C9—C10—C11	121.50 (19)	C3—C8—H8B	109.00
C9—C10—C15	119.75 (18)	C3—C8—H8C	109.00
C11—C10—C15	118.75 (17)	H8A—C8—H8B	109.00
C10—C11—C12	120.5 (2)	H8A—C8—H8C	109.00
C11—C12—C13	119.7 (2)	H8B—C8—H8C	109.00
Cl1—C13—C12	119.21 (17)	N1—C9—H9	119.00
Cl1—C13—C14	119.62 (18)	C10—C9—H9	119.00
C12—C13—C14	121.17 (19)	C10—C11—H11	120.00
C13—C14—C15	119.3 (2)	C12—C11—H11	120.00
C10—C15—C14	120.7 (2)	C11—C12—H12	120.00
C3—C4—H4	119.00	C13—C12—H12	120.00
C5—C4—H4	119.00	C13—C14—H14	120.00
C4—C5—H5	120.00	C15—C14—H14	120.00
C6—C5—H5	120.00	C10—C15—H15	120.00
C1—C6—H6	120.00	C14—C15—H15	120.00
			
C9—N1—C1—C2	139.41 (17)	C3—C4—C5—C6	0.9 (4)
C9—N1—C1—C6	−44.1 (2)	C4—C5—C6—C1	−0.6 (4)
C1—N1—C9—C10	176.57 (15)	N1—C9—C10—C11	−6.5 (3)
N1—C1—C2—C3	178.22 (15)	N1—C9—C10—C15	172.75 (18)
N1—C1—C2—C7	−3.0 (2)	C9—C10—C11—C12	178.72 (18)
C6—C1—C2—C3	1.7 (3)	C15—C10—C11—C12	−0.5 (3)
C6—C1—C2—C7	−179.55 (19)	C9—C10—C15—C14	−178.54 (19)
N1—C1—C6—C5	−177.17 (19)	C11—C10—C15—C14	0.7 (3)
C2—C1—C6—C5	−0.7 (3)	C10—C11—C12—C13	0.4 (3)
C1—C2—C3—C4	−1.4 (3)	C11—C12—C13—Cl1	179.79 (17)
C1—C2—C3—C8	177.88 (17)	C11—C12—C13—C14	−0.4 (4)
C7—C2—C3—C4	179.87 (19)	Cl1—C13—C14—C15	−179.61 (18)
C7—C2—C3—C8	−0.9 (3)	C12—C13—C14—C15	0.6 (4)
C2—C3—C4—C5	0.1 (3)	C13—C14—C15—C10	−0.7 (3)
C8—C3—C4—C5	−179.1 (2)		

### Hydrogen-bond geometry (Å, °)

**Table:**

Cg1 and Cg2 are the centroids of the C1–C6 and C10–C15 benzene rings, respectively.

**Table:**

*D*—H···*A*	*D*—H	H···*A*	*D*···*A*	*D*—H···*A*
C6—H6···Cg1^i^	0.93	2.99	3.649 (2)	129
C7—H7A···Cg2^ii^	0.96	2.93	3.757 (3)	145
C12—H12···Cg1^iii^	0.93	2.96	3.793 (3)	150
C7—H7E···Cg2^ii^	0.96	3.00	3.757 (3)	137

Symmetry codes: (i) −*x*+1, *y*+1/2, −*z*+1/2; (ii) −*x*+1, −*y*, −*z*; (iii) −*x*+1, −*y*+1, −*z*.
